# Exploring the Longitudinal Relationship Between Lockdown Policy Stringency and Public Negative Emotions Among 120 Countries During the COVID-19 Pandemic: Mediating Role of Population Mobility

**DOI:** 10.3389/fpsyt.2022.753703

**Published:** 2022-05-10

**Authors:** Weigang Gong, Guodong Ju, Meng Zhu, Senhu Wang, Wei Guo, Yunsong Chen

**Affiliations:** ^1^Department of Sociology, Wuhan University, Wuhan, China; ^2^Department of Social Policy, London School of Economics and Political Science, London, United Kingdom; ^3^The Institute for Advanced Studies in Finance and Economics, Hubei University of Economics, Wuhan, China; ^4^Department of Sociology, National University of Singapore, Singapore, Singapore; ^5^School of Social and Behavioral Sciences, Nanjing University, Nanjing, China; ^6^The Centre for Asia-Pacific Development Studies, Nanjing University, Nanjing, China

**Keywords:** COVID-19, lockdown policies, public emotions, population mobility, public health policies

## Abstract

**Background:**

To limit the spread of COVID-19, governments worldwide have implemented a series of lockdown policies to restrict the social activities of people. Although scholars suggest that such policies may produce negative effects on public emotions, the existing research is limited because it only provides a cross-sectional snapshot of the effect of lockdown policies in small and local samples. Using large-scale longitudinal cross-country data, the current study aims to gain a better understanding of the dynamic effect of lockdown policies on public emotions and their underlying mechanisms.

**Methods:**

Drawing on a large-scale longitudinal data from multiple sources, the study employs fixed-effects models to analyze the association between lagged lockdown policy stringency and public negative emotions among 120 countries from February to July 2020 (*N* = 9,141 country-day observations). The bootstrapping mediation test is used to examine the mediation effects of increased population mobility in residential areas.

**Results:**

The results show a statistically significant and positive association between lagged lockdown policy stringency and general public negative emotion (standardized coefficient = 0.32, CI = 0.30–0.35, *p* < 0.001). This pattern remains similar to other specific negative emotions, such as depression, anxiety, hopelessness, and helplessness. Moreover, the negative health effects of lockdown policy stringency are significantly mediated by increased mobility in residential areas (51–74% points, *p* < 0.001).

**Conclusion:**

The findings confirm that stringent lockdown policies have a negative effect on public emotions via confining population mobility residential areas. To tackle the COVID-19, future public health policies should pay more attention to the unintended negative consequences of lockdown measures on public emotions.

## Background

As a novel pandemic officially declared by the World Health Organization (WHO) on March 12, 2020, COVID-19 has spread across the globe with an exponential increase of affected cases (1, 2). Until the end of February 2021, the number of confirmed cases of COVID-19 worldwide reached approximately 112 million, which includes nearly 2.5 million deaths (3). With such a severe situation, many countries declared stringent policies to constrain people’s mobility and flatten the epidemic curve, which holds significant implications for public health. Although ample research has evaluated the necessity and effectiveness of such policies to reduce the infection rate of the pandemic (4, 5), relatively scarce attention has been paid to the effects of such policies on public emotions. This aspect prevents governments and scholars from comprehensively understanding the impacts of policies to enable appropriate adjustments.

Taking a retrospective perspective, prior studies have illuminated that there is a substantial negative association between stringent lockdown policies and public mental health during a public crisis. For instance, Wu et al. compared the mental condition of health workers who have been quarantined during the SARS with those who have not and found that those who endured the quarantine were 2–3 times more likely to suffer from posttraumatic stress than their colleagues (6). Moreover, Jeong et al. investigated the impact of being isolated on people’s mental status during the MERS in Korea. They found that even at 4–6 months after they were released, anxiety symptoms derived from such experience can still be observed (7). Other research is also in line with the finding that there are significant negative effects on mental health conditions due to official quarantine policies and such effects can range from immediate impacts to long-term results (7–9).

Regarding the COVID-19 that we are facing, ample empirical research also backs up this association. Studies reveal that the level of distress or anxiety in population can significantly grow during a pandemic (10). Among the limited discussions, Qiu et al. found that many respondents suffered from anxiety symptoms or psychological distress during the COVID-19 pandemic in China (11). A study conducted in Kolkata of India further found that many people living with depression during COVID-19 pandemic were also experiencing an increased sense of helplessness and hopelessness about the future (12). Herat explored the impact of lockdown using a linguistic approach to analyze newspaper contents and suggested that people felt more anxious during the quarantine (13). Casagrande et al. conducted an online survey based on Italian people affected by lockdown restrictions (14). The study demonstrated that a majority of respondents reported worse quality of sleep and higher levels of distress compared with the time before the quarantine. Similarly, De Vos also called for attention on the research on the negative impact of social distancing policies on personal wellbeing (15). Gupta et al. comprehensively summarized that lockdown programs can cause pervasive anxiety, distress, and other negative emotions by augmenting the sense of aloneness (16). Furthermore, prior researchers have also identified several indicators that make the quarantine experience to become a stressor for people’s emotion and mental health, which include anxiety, depression, hopelessness, and helplessness (17).

This study draws on Cacioppo’s social isolation theory, which argues that social isolation could activate the neurobiological mechanisms that lead to people’s self-preservation in the short-term and undermine their mental health and increase the rates of morbidity and mortality in the long-term (18, 19). Based on a wide range of experimental and observational studies in epidemiology, the social isolation theory argues that social interaction is a crucial condition for human beings’ survival and prosperity. Specifically, substantial research demonstrates that social isolation has large negative impacts of on physical and mental health, which are equivalent to the effects of smoking, sedentary lifestyle, hypertension and obesity (18, 19). In contrast, ample evidence suggests that regular interactions in various social settings such as workplace are shown to reduce mental stress and thus improve people’s health and wellbeing (20, 21).

**FIGURE 1:**
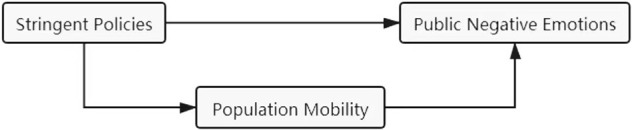
Theoretical framework.

Therefore, in the present study, we pay particular attention to the impact of social isolation among those factors of making the quarantine experience a stressor for people’s emotion and mental health. That is to say, the effect on people’s psychological situation due to the limitation of mobility in their daily life is directly caused by the quarantine and closely associated to social isolation and loneliness. A plausible analogy for this factor is incarceration, which has been well researched by scholars. Evidence shows that when people are confined in a limited space, such a dramatic change can lead to a negative repercussion for people’s mental health by decreasing their connections with others and increasing their stress and anxiety (22). While compared with incarceration, people do not need to meet violence and lack of privacy under the quarantine, the loss of mobility to the outside still serves as a concern for the public emotion. Thus, we theoretically consider that the stringent policy increases people’s negative emotions, which triggers the concern of public mental health, and the pathway is partially mediated by the reduction of people’s mobility in their daily life (Figure 1).

## Current Study

Using large-scale longitudinal data from 120 countries from January to July 2020 (*N* = 9,141 country-day observations), the study aims to fill important gaps in the previous research and achieve two research objectives. First, while a large number of existing studies applied the snowball sampling strategy to recruit online respondents, or focused on particular groups of people (23–27), this study aims to analyze the longitudinal and dynamic association between lagged lockdown policy stringency and public negative emotions, thereby improving the external validity, and providing more comprehensive policy implications. Second, while previous studies have primarily focused on the association between lockdown and public emotions but did not explore the mechanisms, this study aims to test population mobility as a mechanism underlying the relationship between the lockdown policy and people’s negative emotions, deepening our understanding of how worldwide lockdown measures affect public emotions.

## Materials and Methods

### Measures

#### Dependent Variable

The study uses country-level negative emotions as the dependent variable. Data are derived from the Global Database of Events, Language and Tone (GDELT) (28).^1^ It is one of the largest open-access spatio-temporal datasets that identifies the people, organizations, themes, and events that drive society in broadcast, print, and web news in over 100 languages on a daily basis. To measure country-level negative emotions, the study derives cross-national longitudinal data from GDELT related to four keywords, namely, anxiety, depression, hopelessness, and helplessness. The indexes of the four keywords were calculated based on the frequency of occurrence in relevant in broadcast, print, and web news. The four keywords were selected because they have been widely used in mental health research (17). We have also tested other emotional keywords such as sadness and impatience and yielded similar results. Additionally, the four items were combined to construct a single measure of country-level negative emotion using principal component factor analysis. Analyses indicate that the four items display a high level of internal consistency and can be combined as a single indicator of negative emotion (α = 0.86; Kaiser–Meyer–Olkin’s measure of sampling adequacy = 0.795; Bartlett’s test of sphericity: *p* < 0.001; eigenvalue = 2.88; proportion of variance explained = 0.72). The predicted factor score is standardized with a high score indicating strong negative emotions. Thus, the outcome variables consist of the overall negative emotions and their four sub-indicators.

In addition, to test whether GDELT negative emotions can reflect actual negative emotions in the society, we have selected one GDELT negation emotion i.e., “anxiety” and compare it with the “anxiety” index from Twitter (a more direct measure of emotion) in the US in 2020. The Twitter anxiety is measured in a similar way as GDELT and calculated based on 124,118,057 tweets from more than 2 million people in the US in 2020. Overall, we find that the trends of both anxiety measures are very similar throughout the year of 2020 (results available upon request). Furthermore, we have conducted fixed effects models to formally test the relationship between GDELT anxiety and Twitter anxiety, and the impact of lockdown policy stringency on Twitter anxiety in the US. Reassuringly, we find that the relationship between both measures is highly statistically significant (coefficient = 0.21, SE = 0.04, *p* < 0.001) and lockdown policy stringency can also significantly predict Twitter anxiety in the US (coefficient = 0.16, SE = 0.05, *p* = 0.001). These results confirm the validity of GDELT data. However, due to limits of time and funding, a more comprehensive validation of GDELT data is beyond the scope of this article. Thus, we suggest future research to conduct a formal validation of GDELT by comparing it with data from various sources in multiple countries.

#### Independent Variable

The key independent variable is the country-level lockdown policy stringency index, which is derived from the Oxford COVID-19 Government Response Tracker (OxCGRT) (29). It provides a systematic cross-national longitudinal measure of central governments’ policy responses toward COVID-19 across a standardized series of indicators. Furthermore, it creates a suite of composites indices to measure the extent of these responses. The stringency index measures the strictness of central government lockdown policies that restrict people’s behavior, such as closure of schools, workplaces, and public transport, cancelation of public events, implementation of stay-at-home requirements, and restrictions on gathering size, internal movement, and international travel across the country. To consider the temporal order between governmental policies and negative emotions among societies, the study uses the 1-day lagged value of the standardized stringency index, where a high score indicates high policy stringency.

#### Mediators

The study employs cross-national longitudinal data from the Google COVID-19 Community Mobility Reports (GCCMR) to construct the measures for country-level population mobility as the mediator (30). The GCCMR data contain percent changes in number of people in residential areas. These data are compared with the baseline day, which is the median value from the 5-week period from January 3 to February 6, 2020. Therefore, the study uses population mobility change in residential areas to examine a crucial mechanism underlying the effects of lockdown policy stringency on negative emotions.

#### Other Covariates

To eliminate confounding effects from the omitted variables, the study controls for numerous additional OxCGRT governmental policy responses. They include testing policy (four categories, namely, “no testing,” “testing of key workers with symptoms,” “testing of anyone with symptoms,” and “public testing”), contact tracing (three categories, namely, “no contact tracing,” “limited contact tracing,” and “comprehensive contact tracing”). In addition, the study controls for logged COVID-19 cases and death rates per country. To take the effects of time into account, the study further controls for logged time (days) elapsed since the first reported case in each country. Table 1 provides the descriptive statistics for each variable in detail.

**TABLE 1:** Sample characteristics.

		*M*, %	*SD*	Min	Max
Negative emotions (M)		0.09	0.98	−2.75	9.69
Anxiety (M)		0.06	0.97	−2.48	30.56
Depression (M)		0.08	0.98	−2.73	9.57
Helplessness (M)		0.09	0.99	−2.49	12.08
Hopelessness (M)		0.06	0.96	−2.69	7.86
Stringency index (M)		0.34	0.98	−1.88	1.34
Public place mobility (M)		−0.44	0.97	−2.87	2.43
Residential mobility (M)		0.42	0.98	−1.82	3.73
**Testing policy (%)**					
No testing		6.16			
Testing of key-workers with symptoms		53.72			
Testing of anyone with symptoms		29.43			
Public testing		10.69			
**Contact tracing**					
No tracing		18.5			
Limited tracing		35.88			
Comprehensive tracing		45.62			
Logged case ratio		3.96	2.43	0.00	9.59
Logged death ratio		1.37	1.63	0.00	6.69
Logged time since first reported case		3.65	0.81	0.00	4.89
**Number of country-date observations = 9,197**					
**Number of countries = 120** **Country distribution**					
Africa	26	North America		17	
Asia	32	Oceania		4	
Europe	31	South America		10	

*M, Means; %, Proportions.*

#### Analytic Strategy

To analyze the relationship between lockdown policy stringency and society negative emotions, the study uses the fixed effects model, which can be derived as follows:


$$N\,e\,g\,a\,t\,i\,v\,e\,\_\,e\,m\,o\,t\,i\,o\,n\,s_{i_{t}}=\beta_{1}\,S\,t\,r\,i\,n\,g\,e\,n\,c\,y\,\_\,i\,n\,d\,e\,x_{i_{\left(t-1\right)}}+\beta_{2}\,P\,o\,p\,u\,l\,a\,t\,i\,o\,n\,\_\,m\,o\,b\,i\,l\,i\,t\,y_{i_{t}}+\beta_{3}\,C\,o\,n\,t\,r\,o\,l_{i_{t}}+c_{i}+\mu_{i\,t},$$

where *Negative*_*emotion*_*s_i_t__*_ is the dependent variable that represents the level of prevalence of depression in country *i* at time point *t*, *Stringency*_*index*_*i*_(*t*−1)_ denotes the 1-day lagged value of governmental policy stringency index, and *Population*_*mobility*_i_*t*___ stands for the percentage change in population mobility compared with the baseline day in country *i* at time point *t*. In addition, *c_i_* refers to the country-level time constant error term (which will be eliminated during analysis), and μ_*it*_ pertains to other country-level time varying error term. Using the fixed effects model, the researchers ruled out the confounding effects from time invariant country-level factors. In other words, using fixed effects models enables researchers to examine how changes in national lock down policies affect changes in public emotions within countries, achieving a more robust causal inference. Next, we use Hayes’ bootstrapping mediation method (31) to examine the extent to which population mobility in residential areas can explain the effects of lockdown policy stringency on negative emotions (31). We estimate the models using Stata software (release 14).

## Results

### Descriptive Analyses

**FIGURE 2:**
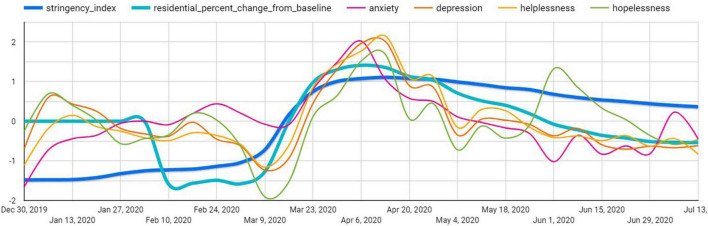
Time series patterns of negative emotion, lockdown stringency index, mobility in public and residential areas.

Figure 2 shows the time series patterns of negative emotion, lockdown stringency index, population mobility in residential areas between February and July 2020. Overall, it shows that since February the lockdown stringency, public negative emotion and population mobility in residential areas gradually increased and peaked in April. From April to July, the trends of increase in negative emotion, lockdown stringency and population mobility in residential areas slowed. While the level of negative emotion decreased to the similar level in February, the levels of lockdown stringency and population mobility in residential areas still remained higher than those in February. Taken together, these patterns suggest that there may be a positive relationship between lockdown stringency and negative emotion, and such association could potentially be mediated by increased population mobility in residential areas.

Further descriptive analyses on country differences (see Supplementary Figures 1–6) generally show that countries that implemented relatively stringent lockdown policies (e.g., South American, some Asian, and African countries) experienced greater increases in population mobility in residential areas compared with countries, which implemented relatively loose lockdown policies (e.g., Scandinavian countries and Japan). However, the link between lockdown policy and negative emotion between countries is not as clear as the time series pattern. For example, while some countries such as India that implemented strict lockdown policies experienced strong negative emotions, other countries such as Scandinavian countries and the USA that implemented relatively loose lockdown policies also experienced strong negative emotions. This suggests that the relationship between lockdown policy and negative emotions is complex and likely to be affected by country-level characteristics such as social institutions, culture, and COVID-19 infection rate. For example, in countries with relatively loose lockdown policies, the higher infection rate could also lead to negative public emotions. Thus, it is important to use fixed-effects models to exclude these country-level confounders.

### Fixed Effects Models

Table 2 shows several fixed effect models examining the lagged effects of the lockdown stringency index on five types of country-level negative emotions during the COVID-19 pandemic. Models 1 shows that the lagged effects of the lockdown stringency index had significantly positive effects on overall negative emotions (*p* < 0.001). Specifically, an increase of one in the standard deviation of the stringency index correlated to an increase of 0.32 standard deviation in the value for negative emotions. A similar pattern is observed for the other four sub-indicators of overall negative emotions, including anxiety, depression, hopelessness, and helplessness. The associations between the stringency index and these four specific negative emotions are statistically significant (*p* < 0.001) with the effects ranging from 0.22 to 0.34. In Supplementary Table 1, we have tried different lag options of policy stringency index. We find that with the increase of time lag, the size and significance of coefficients decrease, and 20 days lagged policy stringency has little impact on negative emotions.

**TABLE 2:** Fixed effects models examining the lagged effects of lockdown policy stringency on negative emotions during the COVID-19 pandemic.

	Negative				
	Emotion	Anxiety	Depression	Helplessness	Hopelessness
Lagged stringency index	0.32***	0.23***	0.32***	0.34***	0.22***
	(0.02)	(0.02)	(0.02)	(0.02)	(0.02)
**Contact tracing (Ref. = No tracing)**					
Limited tracing	−0.06	0.01	−0.00	−0.05	−0.12**
	(0.05)	(0.05)	(0.05)	(0.05)	(0.05)
Comprehensive tracing	0.04	0.05	0.08	0.04	−0.02
	(0.04)	(0.05)	(0.05)	(0.05)	(0.04)
**Testing policy (Ref. = No testing)**					
Testing of key-workers with symptoms	−0.03	−0.12*	−0.02	−0.00	0.01
	(0.05)	(0.06)	(0.05)	(0.05)	(0.05)
Testing of anyone with symptoms	−0.16**	−0.15**	−0.15**	−0.16**	−0.10*
	(0.05)	(0.06)	(0.06)	(0.06)	(0.05)
Public testing	−0.14*	−0.12	−0.13*	−0.16*	−0.07
	(0.06)	(0.07)	(0.06)	(0.06)	(0.06)
COVID-19 death rate	0.17	1.53**	−0.19	−0.54	0.36
	(0.46)	(0.51)	(0.48)	(0.48)	(0.46)
COVID-19 case rate	0.07*	0.17***	0.03	0.04	0.04
	(0.03)	(0.03)	(0.03)	(0.03)	(0.03)
Logged time (days)	−0.07***	−0.14***	−0.05***	−0.05***	−0.02
	(0.01)	(0.02)	(0.01)	(0.01)	(0.01)
Constant	0.22	−0.09	0.28	0.45*	0.00
	(0.20)	(0.23)	(0.21)	(0.21)	(0.20)
Country-date observations	9,197	9,197	9,197	9,197	9,197
Number of countries	120	120	120	120	120
Within R-squared	0.06	0.03	0.06	0.06	0.03

Standard errors are in parentheses. ***p < 0.001, **p < 0.01, *p < 0.05 (two-tailed tests).

In addition, Supplementary Table 2 tests other negative emotions such as sadness and impatience and yields similar results. This suggests that our results are robust to alternative variable specification. Also, as public emotions may change randomly, the relationship between lockdown policy stringency and public emotions may be due to random errors. Thus, we have generated a variable X with random values and examined its relationship with our independent variables as a placebo test in Supplementary Table 2. Overall, we find that there are no statistically significant associations between all explanatory variables and X. This suggests that the relationship between lockdown policy stringency and public emotions may not be due to random errors. Overall, these results suggest that stringent government lockdown policies may lead to a rise of negative emotions in society.

Next, Table 3 shows a number of bootstrapping mediation models (31), which examine the extent to which population mobility in residential areas can explain the effects of lockdown policy stringency on negative emotions. In terms of the overall negative emotion, we find that the indirect effect (i.e., the effect of lockdown policy stringency on negative emotion via population mobility in residential areas) is 0.21 and statistically significant (*p* < 0.001) with confidence intervals ranging from 0.18 and 0.24. Overall, around 65% of total effect is mediated by population mobility in residential areas. The pattern remains similar for other negative emotions including anxiety, depression, helplessness, and hopelessness. For all the four indicators, the indirect effects range from 0.12 to 0.22 and are statistically significant (*p* < 0.001). Around 51–75% of total effects are mediated by population mobility in residential areas for the four negative emotions. Taken together, the results show that high population mobility in residential areas play an important role in explaining the effects of lockdown policy stringency on negative emotions.

**TABLE 3:** Bootstrapping method examining the mediation effects of population mobility in residential areas in the relationship between lockdown policy stringency and negative emotions.

	Negative emotion	Anxiety	Depression	Helplessness	Hopelessness
Total effects	0.32***	0.23***	0.32***	0.34***	0.22***
	(0.30, 0.35)	(0.20, 0.26)	(0.29, 0.35)	(0.31, 0.37)	(0.18, 0.25)
Direct effects	0.11***	0.11***	0.11***	0.12***	0.06**
	(0.07, 0.15)	(0.07, 0.15)	(0.07, 0.15)	(0.07, 0.17)	(0.02, 0.09)
Indirect effects	0.21***	0.12***	0.21***	0.22***	0.16***
	(0.18, 0.24)	(0.08, 0.15)	(0.18, 0.24)	(0.18, 0.25)	(0.13, 0.19)
Percent mediated by PMRA	65.46%	51.15%	67.75%	65.05%	74.57%
Country-date observations	9,197	9,197	9,197	9,197	9,197
Number of countries	120	120	120	120	120

PMRA, population mobility in residential areas. All mediation analyses control for testing policy, contact tracing, COVID-19 case and death rates, and logged time (days) elapsed since the first reported case in each country. Confidence intervals are in parentheses, ***p < 0.001, **p < 0.01 (two-tailed tests).

## Discussion

The COVID-19 pandemic, together with many other factors, has led to increased adverse mental health issues in various countries (32, 33). To the best of our knowledge, this is the first study to provide substantial evidence concerning the effects of lockdown policies on people’s emotions while also deepening our understanding of the underlying mechanisms associated with these emotions based on large-scale, longitudinal cross-country data. In conclusion, this study yields two important findings.

Consistent with previous studies in Nepal, Italy, and India that used small and localized samples (16, 32, 34), our study further confirms that stringent lockdown policies could have a strong negative correlation on a range of public emotions, such as anxiety, depression, hopelessness, and helplessness. In addition, this study extends the previous literature on the relationship between lockdown policies and negative emotions in two important ways. First, this study used longitudinal data and lagged variable models to demonstrate that the negative impact of lockdown policies can last about 20 days, revealing the dynamic relationship between lockdown policies and negative emotions. Second, by analyzing data in 120 countries this study extends beyond any particular countries or population groups, and thus can be generalized to other countries and holds significant implications for the global pandemic response-lockdown policies designed to limit the spread of the COVID-19 but may have other negative and unintended consequences on people’s emotions. While tackling the COVID-19 pandemic, future public health policies should take into account the negative spillover effects of lockdown measures (35).

As another contribution to the previous literature, this study revealed a crucial mechanism underlying the negative impact of lock-down policies by demonstrating that the negative effects of lockdown policies can be significantly mediated by increased mobility in residential areas. This suggests that changes in population mobility in residential areas is an important mechanism through which lockdown policies lead to negative emotions among the public. Previous studies in Nordic and South-European countries have reported that the ability and opportunity to spend time outdoors may be especially important under conditions of lockdown and social-distancing, and long-time stay-at-home could lead to mental health problems (36, 37). In the present study, the finding is overall consistent with the previous studies and suggests that people who stay at home or spend a considerable amount of time in residential areas tend to develop depression, loneliness, and other mental health problems (38). However, there is still a sizable proportion of the effect that cannot be mediated by changes in population mobility. This is likely due to the direct effect of lockdown policies on people’s emotions, as well as other potential mediators, such as changes in lifestyles and working environments.

To summarize, this study makes two important contributions to the previous research. First, our study is based on a panel dataset that covers about 120 countries in 4 months, which allows us to expel unobservable confounders and identify the causal relationship. In contrast, a large number of existing studies applied the snowball sampling strategy to recruit online respondents, distributed online surveys on social network, or focused on particular groups of people, such as medical workers, children, and senior citizens in particular countries or regions (23–27). Second, previous studies have primarily focused on the association between lockdown and public emotions but did not explore the mechanisms. This may limit the in-depth and comprehensive understanding of how lockdown measures affect public emotions. We propose a clear mechanism underlying the relationship between the lockdown policy and people’s negative emotions. Specifically, we argue that the negative impacts of lockdown on negative emotions could be partially explained by people’s restricted mobility. Thus, this study fills an important research gap by demonstrating how lockdown measures affect public emotions.

This study also has some limitations that should be further explored in future research. First, the results of this research are based on country-level data and thus cannot be generalized to the individual level for the potential ecological fallacy. It would be advantageous for future research to combine individual- and country-level data in order to examine the health effects of lockdown policies. Second, this study only tested the mediation effects of population mobility. There may be other mechanisms through which lockdown measures could influence public emotions, such as decreased social contact or changes in working or neighborhood environments (39, 40). Third, this study only explored the impact of lockdown policies on people’s emotions. Future research could explore the impact of lockdown measures can be moderated by other variables, such as employment status or participation in leisure activities (41, 42). Finally, while this study only focused on public emotions due to limited space, future research could profitably study how lockdown measures affect suicidal risks, health behavior such as alcohol consumption, sedentary lifestyles, or other mental disorders etc.

## Conclusion

The study contributes to the literature in three important ways. First, the study has much higher external validity than previous research and is able to generalize the findings beyond a single country by drawing on a sample of 120 countries. Second, the study helps gain a better understanding of the dynamic relationship between lockdown policies and public emotions by using rich panel data. Third, the study provides important insights into the mechanisms through which lockdown policies affect public emotions by exploring the mediating effects of population mobility. Overall, the findings demonstrate a strong negative effect of stringent lockdown policies on public emotions, thus highlighting the important mediating role of population mobility in public and residential areas. The study calls for further policy attention to the negative unintended consequences of lockdown measures on public emotions.

## Data Availability Statement

Publicly available datasets were analyzed in this study. This data can be found here: Data are derived from the Global Database of Events, Language and Tone (GDELT). It is one of the largest open-access spatio-temporal datasets that identifies the people, organizations, themes, and events that drive society in broadcast, print, and web news in over 100 languages on a daily basis. Further enquires can be directed to the corresponding authors.

## Author Contributions

YC, SW, and WGu led the study and drafted the manuscript. MZ, GJ, SW, WGo, and YC collected, summarized, and analyzed the data. MZ, GJ, SW, and WGu contributed to sections of the manuscript. All authors reviewed and revised the manuscript, read and approved the final manuscript.

## Conflict of Interest

The authors declare that the research was conducted in the absence of any commercial or financial relationships that could be construed as a potential conflict of interest.

## Publisher’s Note

All claims expressed in this article are solely those of the authors and do not necessarily represent those of their affiliated organizations, or those of the publisher, the editors and the reviewers. Any product that may be evaluated in this article, or claim that may be made by its manufacturer, is not guaranteed or endorsed by the publisher.

## Footnotes

1 Further information can be found in the paper (28). Global Database of Events, Language, and Tone (GDELT) and in GDELT website: https://blog.gdeltproject.org/.
