# Supplementary material for: Exploring the Longitudinal Relationship Between Lockdown Policy Stringency and Public Negative Emotions Among 120 Countries During the COVID-19 Pandemic: Mediating Role of Population Mobility
Source: Front Psychiatry. 2022 May 10;13:753703. doi: 10.3389/fpsyt.2022.753703 (PMC9128016; doi:10.3389/fpsyt.2022.753703)
Supplement: Supplementary file 1 [file Data_Sheet_1.docx]

**Online supplementary materials**


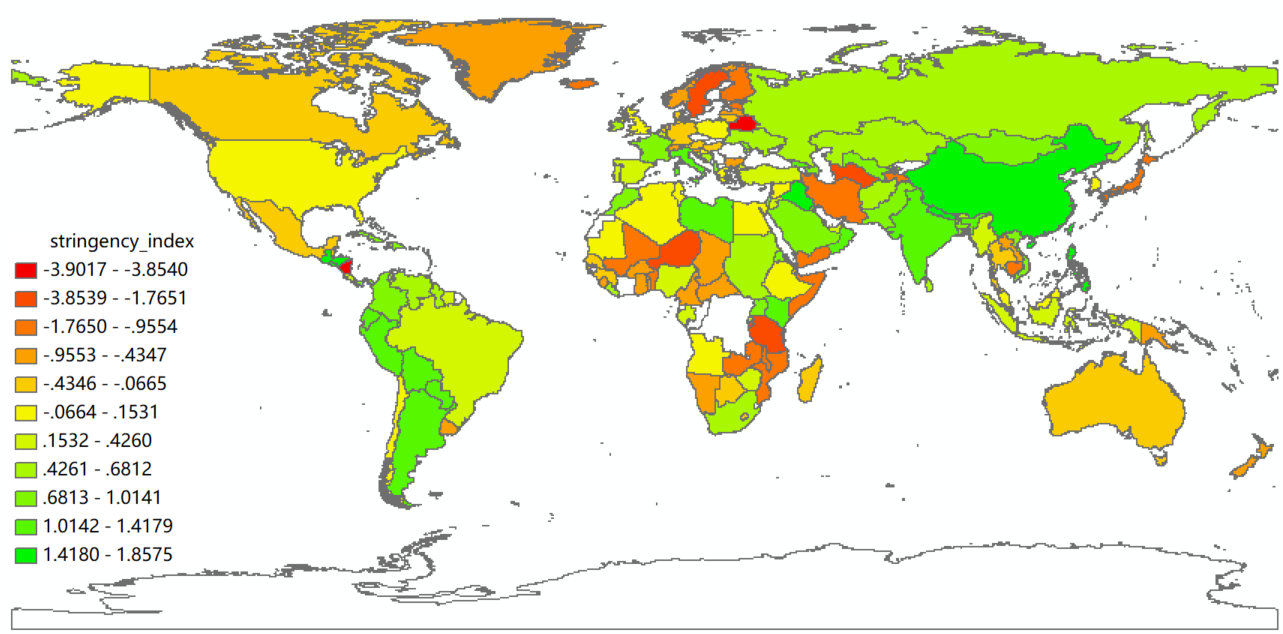


**Figure A1.** Country differences in averaged lockdown stringency index from February to July 2020.


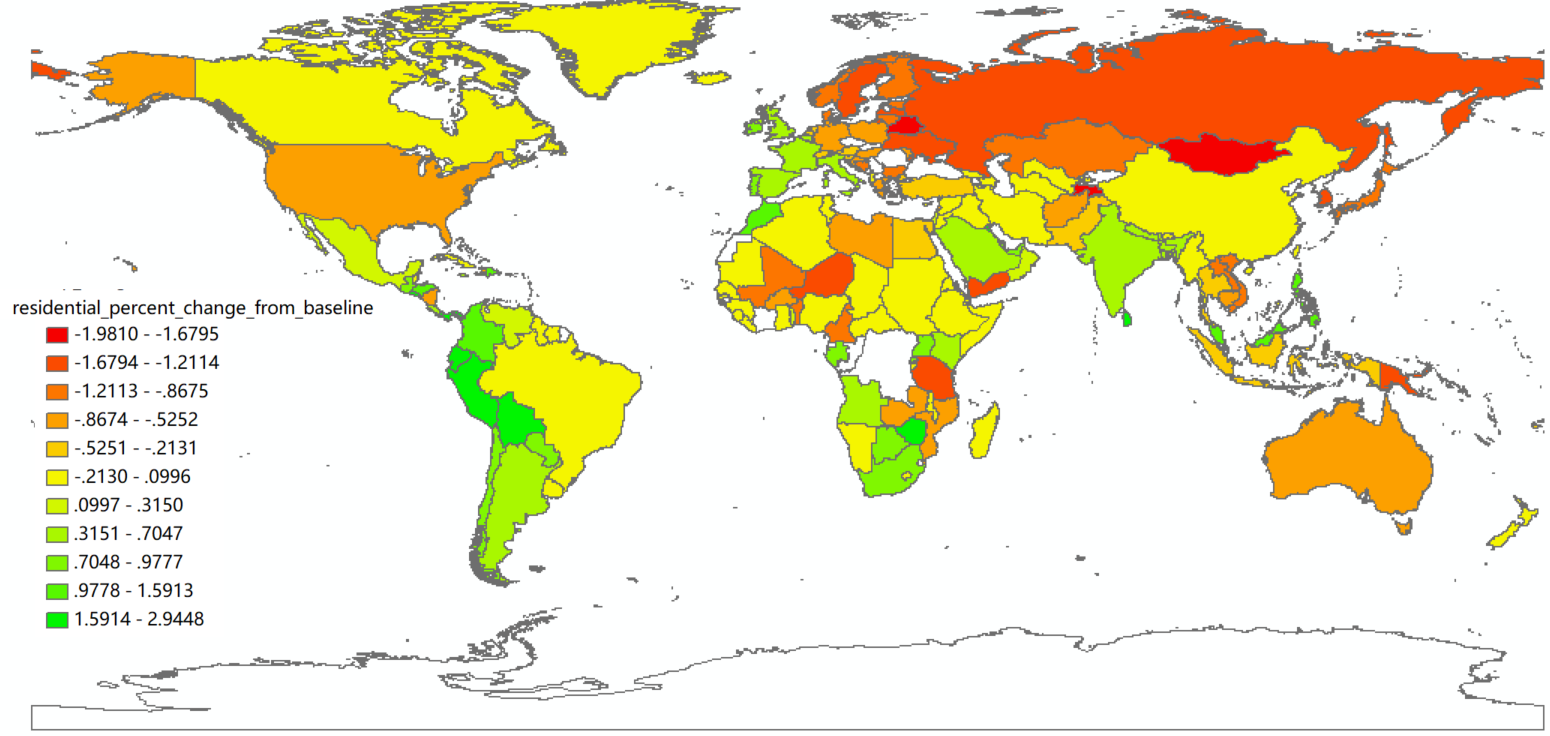


**Figure A2.** Country differences in averaged percent changes of population mobility in residential areas from February to July 2020.


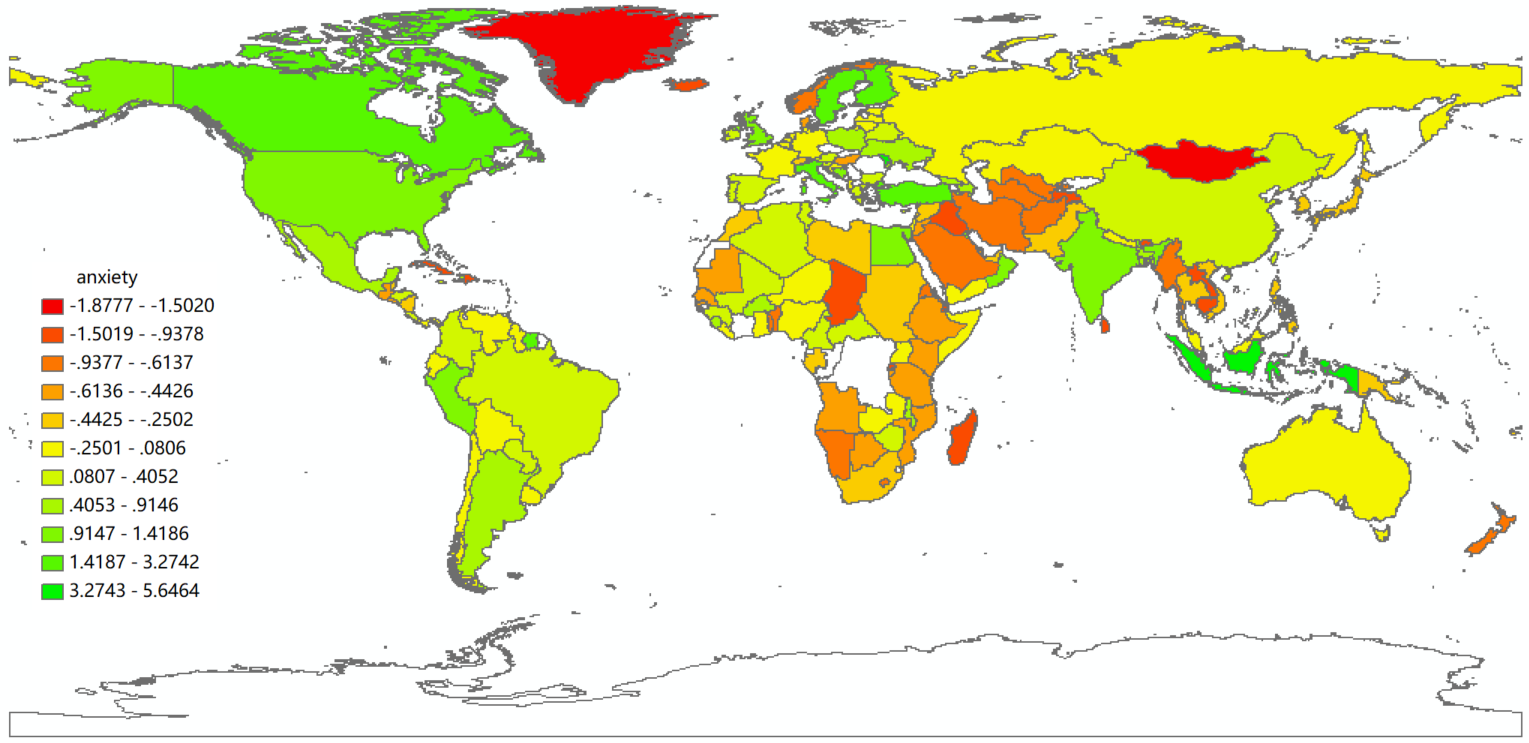


**Figure A3.** Country differences in averaged anxiety score from February to July 2020.


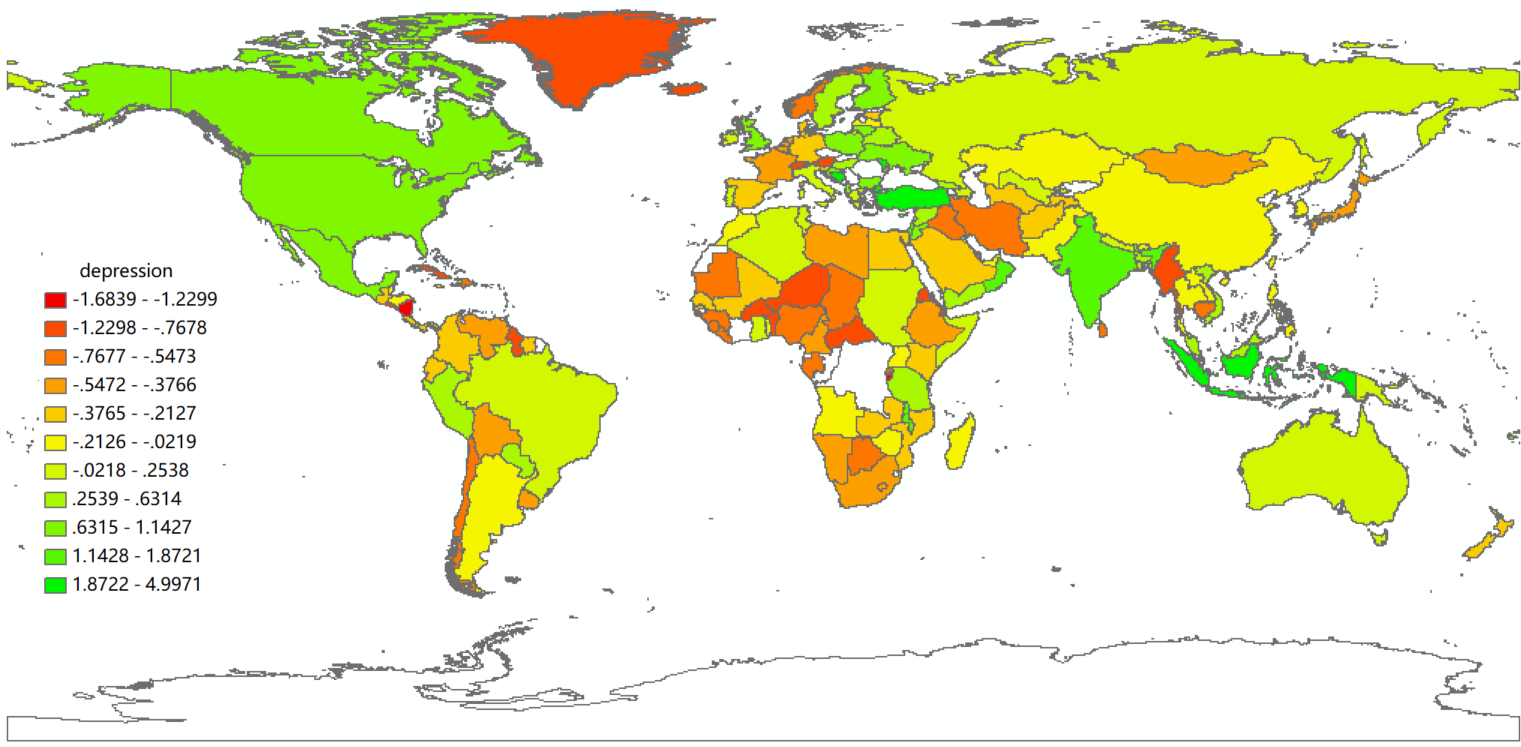


**Figure A4.** Country differences in averaged depression score from February to July 2020.


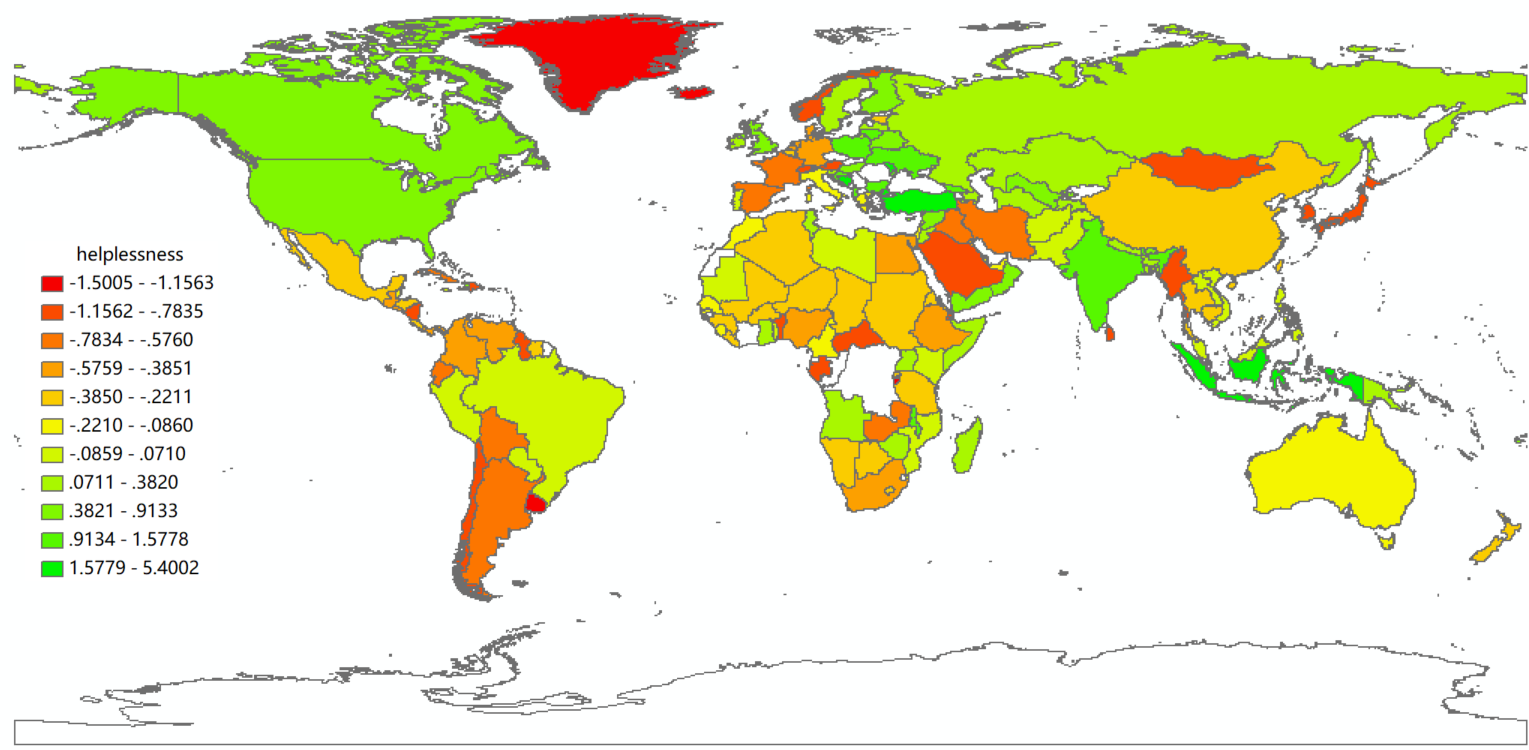


**Figure A5.** Country differences in averaged helplessness score from February to July 2020.


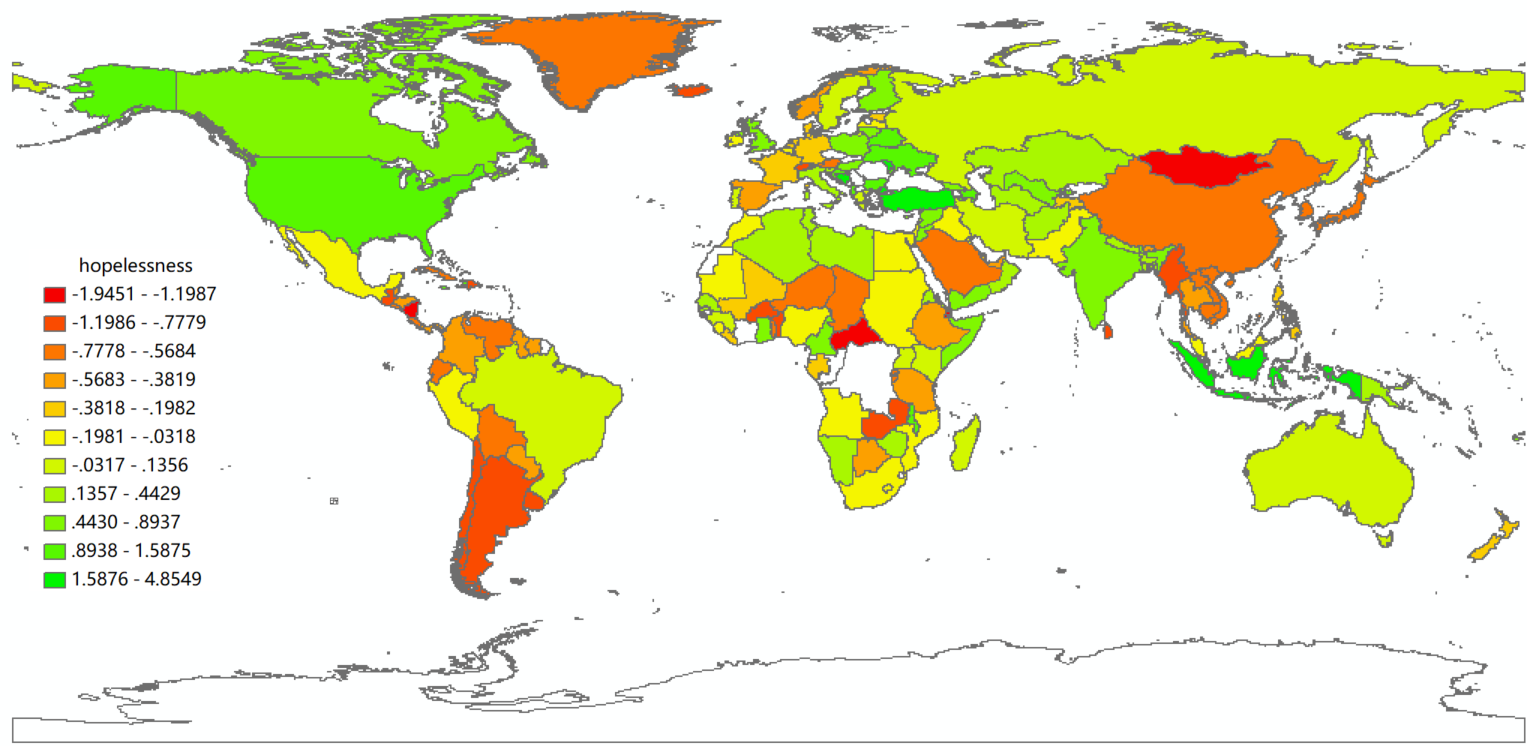


**Figure A6.** Country differences in averaged hopelessness score from February to July 2020.

| **Table A1.** Fixed effects models examining the lagged effects of lockdown policy stringency on country negative emotions during the COVID-19 pandemic (with different lag options). | | | | | |
| --- | --- | --- | --- | --- | --- |
|  | Negative |  |  |  |  |
|  | emotion | Anxiety | Depression | Helplessness | Hopelessness |
| **Models 1** |  |  |  |  |  |
| Lagged stringency index (1 day lag) | 0.32*** | 0.23*** | 0.32*** | 0.34*** | 0.22*** |
|  | (0.02) | (0.02) | (0.02) | (0.02) | (0.02) |
| Country-date observations | 9,197 | 9,197 | 9,197 | 9,197 | 9,197 |
| Number of countries | 120 | 120 | 120 | 120 | 120 |
| **Models 2** |  |  |  |  |  |
| Lagged stringency index (5 days lag) | 0.36*** | 0.24*** | 0.34*** | 0.35*** | 0.27*** |
|  | (0.02) | (0.02) | (0.02) | (0.02) | (0.02) |
| Country-date observations | 8,928 | 8,928 | 8,928 | 8,928 | 8,928 |
| Number of countries | 120 | 120 | 120 | 120 | 120 |
| **Models 3** |  |  |  |  |  |
| Lagged stringency index (10 days lag) | 0.30*** | 0.22*** | 0.28*** | 0.28*** | 0.24*** |
|  | (0.02) | (0.02) | (0.02) | (0.02) | (0.02) |
| Country-date observations | 8,605 | 8,605 | 8,605 | 8,605 | 8,605 |
| Number of countries | 120 | 120 | 120 | 120 | 120 |
| **Models 4** |  |  |  |  |  |
| Lagged stringency index (15 days lag) | 0.19*** | 0.13*** | 0.18*** | 0.17*** | 0.17*** |
|  | (0.02) | (0.02) | (0.02) | (0.02) | (0.02) |
| Country-date observations | 8,268 | 8,268 | 8,268 | 8,268 | 8,268 |
| Number of countries | 120 | 120 | 120 | 120 | 120 |
| **Models 5** |  |  |  |  |  |
| Lagged stringency index (20 days lag) | 0.05** | 0.01 | 0.05** | 0.03 | 0.07*** |
|  | (0.01) | (0.02) | (0.02) | (0.02) | (0.01) |
| Country-date observations | 7,954 | 7,954 | 7,954 | 7,954 | 7,954 |
| Number of countries | 120 | 120 | 120 | 120 | 120 |
| Note. Standard errors are in parentheses. *** p < 0.001, ** p < 0.01, * p < 0.05 (two-tailed tests). | | | | | |

| **Table A2.** Fixed effects models examining the lagged effects of lockdown policy stringency on sadness, impatience and random X during the COVID-19 pandemic | | | |
| --- | --- | --- | --- |
|  | Sadness | Impatience | Random X |
| Lagged stringency index | 0.21*** | 0.20*** | 0.01 |
|  | (0.02) | (0.01) | (0.02) |
| Contact tracing (Ref. = No tracing) |  |  |  |
| Limited tracing | -0.03 | 0.10* | -0.08 |
|  | (0.05) | (0.04) | (0.07) |
| Comprehensive tracing | 0.13** | 0.15*** | -0.06 |
|  | (0.05) | (0.04) | (0.06) |
| Testing policy (Ref. = No testing) |  |  |  |
| Testing of key-workers with symptoms | -0.04 | -0.06 | 0.13 |
|  | (0.05) | (0.04) | (0.07) |
| Testing of anyone with symptoms | -0.13* | -0.06 | 0.14 |
|  | (0.05) | (0.05) | (0.07) |
| Public testing | -0.07 | -0.10 | 0.07 |
|  | (0.06) | (0.05) | (0.08) |
| COVID-19 death rate | 0.46 | 0.02 | 0.14 |
|  | (0.47) | (0.41) | (0.64) |
| COVID-19 case rate | 0.05 | 0.16*** | -0.01 |
|  | (0.03) | (0.03) | (0.04) |
| Logged time (days) | 0.05** | -0.15*** | -0.02 |
|  | (0.02) | (0.01) | (0.02) |
| Constant | -0.39 | 0.52** | -0.07 |
|  | (0.21) | (0.18) | (0.29) |
| Country-date observations | 9,197 | 9,197 | 9,197 |
| Number of countries | 120 | 120 | 120 |
| Within R-squared | 0.05 | 0.03 | 0.00 |
| Note. Standard errors are in parentheses. *** p < 0.001, ** p < 0.01, * p < 0.05 (two-tailed tests). | | | |
